# The efficacy and safety of the Chinese herbal medicine Di-Tan decoction for treating Alzheimer’s disease: protocol for a randomized controlled trial

**DOI:** 10.1186/s13063-015-0716-z

**Published:** 2015-04-30

**Authors:** Ka-Kit Chua, Adrian Wong, Pauline Wing-Lam Kwan, Ju-Xian Song, Lei-Lei Chen, Andrew Lung-Tat Chan, Jia-Hong Lu, Vincent Mok, Min Li

**Affiliations:** School of Chinese Medicine, Hong Kong Baptist University, Kowloon Tong, Kowloon Hong Kong; Stroke and Clinical Neurosciences, Institute of Integrative Medicine, Department of Medicine and Therapeutics, Prince of Wales Hospital, The Chinese University of Hong Kong, Shatin, New Territories Hong Kong; Divisions of Neurology & Geriatrics, Department of Medicine, Queen Elizabeth Hospital, Kowloon, Hong Kong; State Key Laboratory of Quality Research in Chinese Medicine, Institute of Chinese Medical Sciences, University of Macau, Taipa, Macau

**Keywords:** Alzheimer’s disease, Randomized controlled trial, Di-Tan decoction, Chinese medicine

## Abstract

**Background:**

Alzheimer’s disease (AD) is the most common type of dementia in the elderly. It is estimated that the global prevalence of dementia will rise from 24.3 million in 2005 to 81.1 million in 2040. AD has a devastating impact on sufferers, caregivers, their communities and the healthcare system in general. “Di-tan decoction” (DTD) is a traditional Chinese medicine (TCM) formula frequently used to treat symptoms that are now defined as AD in clinical treatment. However, the existing evidence for recommending DTD in clinical practice derives from studies that were methodologically flawed. In this study, we aim to determine the efficacy and safety of DTD in AD patients based on a rigidly randomized controlled trial. It will provide critical information on sample size and treatment regimen for conducting a full-scale clinical trial of DTD later.

**Methods/Design:**

This study will be a double-blind, randomized, placebo-controlled, add-on trial. After a 2-week run-in period, eligible patients with mild to moderate AD will be recruited and given either DTD or placebo twice daily for 24 weeks with follow-up 6 weeks after the last treatment. An increase of four points or greater on the scores of Alzheimer’s Disease Assessment Scale-cognitive subscale (ADAD-cog) will be considered as a positive primary outcome. Total scores of the ADAD-cog, the Chinese version of Mini-Mental State Examination (C-MMSE), and the Chinese version of the Disability Assessment for Dementia (C-DAD) score will be used as secondary outcomes. Adverse events will also be reported.

**Discussion:**

This randomized trial will be the first rigorous empirical study on the efficacy of DTD for treating cognitive symptoms in AD patients. Its success will justify and warrant a large-scale clinical trial to further consolidate the evidence for DTD’s efficacy in treating AD.

**Trial registration:**

Chinese Clinical Trial Registry (ChiCTR-TRC-12004548, Date of registration: 22 November 2012)

**Electronic supplementary material:**

The online version of this article (doi:10.1186/s13063-015-0716-z) contains supplementary material, which is available to authorized users.

## Background

Alzheimer’s disease (AD) is an irreversible, progressive neurodegenerative disorder characterized by loss of cognitive functions, behavioral disturbances and daily living difficulties [1]. It is the most common type of dementia in the elderly [2]. Progressive atrophy is seen in all parts of the brain, especially in the hippocampus region, due to nerve cell death and tissue loss [3]. It is estimated that the number of people suffering from dementia over the world will rise from 24.3 million in 2005 to 81.1 million in 2040 [4]. It is expected that a new case of AD will be diagnosed every 33 seconds in America by 2050 [5].

AD has a devastating impact not only on sufferers, but also on caregivers, their communities and the health care system in general [6]. While much research is being done on many aspects of AD, including its basic biology, no drug has been found to alter the course of the disease [7]. Current pharmacological treatment for AD such as cholinesterase inhibitors (ChEIs) and memantine are symptomatic and may commonly associated with some side effects, such as nausea, vomiting, dizziness and anorexia [8]. Given that the efficacy is mild but with some side effects, patients often seek alternative treatments [9].

In traditional Chinese medicine (TCM), AD is described in terms of TCM principles; it could be caused by (1) a deficiency of vital energy of the kidney, heart and spleen or (2) the stagnation of blood and/or phlegm [10]. The guidelines that classified dementia into different subtypes according to the TCM theory was published in 1990, and symptoms of “phlegm turbidity obstructing the orifices” (PTOO) is considered to be one of the major contributing factors to AD [11]. Thus, the primary TCM strategy for treating AD is to “resolve phlegm in order to open the orifices” [12]. The classic Chinese medicine formula “Di-tan decoction” (DTD) was developed by a famous TCM doctor, Dong Su, in 1449 with the specific function of “resolving phlegm to open the orifices” [13]. It has been and still is frequently used to treat symptoms that are now defined as AD in clinical treatment with TCM [14,15]. In recent years, modern studies have attempted to verify the clinical effects of DTD in biochemical terms. Laboratory studies have shown that the memory impairment of AD model mice was significantly reduced by DTD [16,17]. In the brain tissue in mice treated with DTD, acetylcholine (Ach) and acetylcholine transferase (ChAT) were significantly increased, while acetylcholine esterase (AchE) was decreased [18]. The results of another study indicate that DTD may inhibit the decline of the dopamine content in brain tissue of model mice as well [19].

In our previous systematic review, there was no clinical trial using DTD as the main intervention for treating AD. For the AD clinical trials that had involved DTD as an adjunct intervention, we found that most of them support the efficacy of DTD in treating AD. However, the evidence for recommending DTD in clinical practice derives from studies that were methodologically flawed. None of them was a randomized controlled trial. They not only lacked randomization, blinding, and the use of control groups, but also failed to define inclusion and exclusion criteria and the quality of the intervention medicine [20]. Because of the methodological problems in previous studies, evidence to suggest that DTD is effective in treating AD is not strong. There is a lack of conclusive evidence to recommend a clinical trial.

As there is no basic information of DTD to conduct a comprehensive AD clinical trial, a rigorous pilot study, which focuses on the efficacy and safety of DTD for treating AD, will be needed before conducting a full-scale clinical trial. In this study, we will evaluate the efficacy and safety of DTD in patients with AD in a randomized controlled trial. This study will be a randomized, double-blinded, placebo-controlled study, with restrictive inclusion and exclusion criteria and a clearly defined quality control intervention medicine. We hope the results from this study will provide critical information on sample size and treatment regimen for conducting a full-scale clinical trial later.

## Methods/Design

### Study design

This study will be a double-blinded, randomized, placebo-controlled, add-on trial. Patients with mild to moderate AD will be randomly assigned to receive 24 weeks of either active herbal treatment or placebo (in a 1:1 ratio); they will be followed for a further 6 weeks’ observation period without treatment. This clinical study will be carried out at the Hong Kong Baptist University Chinese Medicine Specialty Centre. It has been approved by the Ethics Committee of the Hong Kong Baptist University’s Institutional Review Board (code: HASC/11-12/24) and registered with the Chinese Clinical Trial Registry (ChiCTR-TRC-12004548). If there is any amendment to the protocol, approval must be again sought from the Ethics Committee. Written informed consent will be obtained from every patient before participation in any study-related activity. The protocol design is based on the guidelines of Consolidated Standards of Reporting Trials (CONSORT) and Standard Protocol Items: Recommendations for Interventional Trials (SPIRIT) [see Additional file 1], and study results will be reported according to these guidelines as well.

### Participants

Inclusion criteria are as follows: Adults who (1) have been clinically diagnosed with AD based on the criteria of National Institute of Neurological and Communicative Disorders and Stroke and Alzheimer’s Disease and Related Disorders Association (NINCDS/ADARA) [21], and (2) present symptoms classified as PTOO [see Additional file 2] as defined by the Guidance for Clinical Research of New Chinese Herbal Medicine [22] during a screening visit, will be eligible. The diagnostic criteria of PTOO include dementia, heavy-headedness and spitting phlegm. Additional inclusion criteria are as follows: (1) mild to moderate dementia with ≥2 on Clinical Dementia Rating Scale (CDR) [23]; (2) receiving a stable dose of anti-dementia medication (that is, Donepezil, Rivastigmine and Memantine) for at least 4 weeks before the start of treatment; and (3) normal liver and renal function.

Exclusion criteria are as follows: Patients who also have any other type of dementia (that is, vascular dementia), and/or other neurodegenerative disorder (that is, Parkinson’s disease), depression (defined by a score of ≥8 on the 15-item Chinese version of the Geriatric Depression Scale [24]), or who are unwilling to cooperate with treatment procedures will be excluded. For those who have participated in other trials within 30 days of the start of this trial as well as women who are pregnant or breastfeeding will also be excluded.

### Study medication

The herbal medicine under study is DTD (Table 1). It is composed of Arisaema Cum Bile (DanNanXing in Chinese), Pinelliae Rhizoma (FaBanXia in Chinese), Aurantii Immaturus Fructus (ZhiShi in Chinese), Poria (FuLing in Chinese), Citri Reticulatae Pericarpium (ChenPi in Chinese), Acori Tatarinowii Rhizoma (ShiChangPu in Chinese), Ginseng Radix (RenShen in Chinese), Bambusae in Taeniam Caulis (ZhuRu in Chinese), Glycyrrhizae Radix (GanCao in Chinese), Zingiberis Recens Rhizoma (ShengJiang in Chinese) and dextin [13]. The test report of the DTD quality control is attached in the Additional file 3. The placebo is made of caramel (2%), gardenia yellow pigment (0.05%), sunset yellow (0.02%), tartrazine (0.02%), dextrin (95%) and broadleaf holly leaf (2.91%) [25]. The granules were produced in a single batch (DTD batch no.: A12074 Placebo batch no.: A120817) strictly in compliance with standards of Good Manufactory Practice (GMP) and Chinese Pharmacopoeia 2010 to ensure the stability and homogeneity of the composition as produced by PuraPharm Pharmaceuticals Company Limited. The chemical compositions of the final products were analyzed for contamination with heavy metals, toxic elements, microbe and pesticide residues; both final products were analyzed for stability and adherence to quality standards. The active treatment granules and the placebo granules have identical appearance and smell, and both have been packed in sealed opaque aluminum sachets and put in zip lock bags (10 sachets each). Only the treatment code is printed on the package to ensure successful blinding of patients [26]. One sachet contains 13.5 g, a dosage equivalent to 67.5 g herbs. All herbal and placebo granules will be distributed by L.L. Chen with both written and verbal instructions for each participant. Patients will be instructed to take the granules orally by dissolving a sachet of granules in 150 ml hot water, stirring well, then drinking the solution, two times per day, at least two hours apart from taking any routine Western medication. Patients will be allowed to discontinue the study granules temporarily if any adverse events occur. Patients will be instructed to report such events to a special e-mail account or by a direct telephone line. Additional treatments for a newly occurring illness, for example, flu or diarrhea, or adverse event during the study will be allowed but must be reported to the assessor.

**Table 1:** **“Di-tan decoction” (DTD) composition**

**Chinese name**	**Latin name**	**Percentage**
Dan Nan Xing	Arisaema Cum Bile	11.42
Ban Xia	Pinelliae Rhizoma	11.42
Zhi Shi	Aurantii Immaturus Fructus	9.13
Fu Ling	Poria	9.13
Chen Pi	Citri Reticulatae Pericarpium	6.84
Shi Chang Pu	Acori Tatarinowii Rhizoma	4.55
Ren Shen	Ginseng Radix	4.55
Zhu Ru	Bambusae in Taeniam Caulis	3.19
Gan Cao	Glycyrrhizae Radix	2.29
Sheng Jiang	Zingiberis Recens Rhizoma	18.10
Dextrin	*-*	19.36

### Recruitment procedures

Three methods will be used to recruit participants with AD. The first source of candidates will be referral from two public Western medicine hospitals in Hong Kong (Prince of Wales Hospital and Queen Elizabeth Hospital) from our co-investigator (Co-I), V. Mok, and our research teammate, A.L.T. Chan. The second source will be referral from the Chinese medicine clinic of Hong Kong Baptist University. A third source of candidates will be those who respond to advertisements published in local newspapers and newsletters of local AD societies.

**Figure 1:**
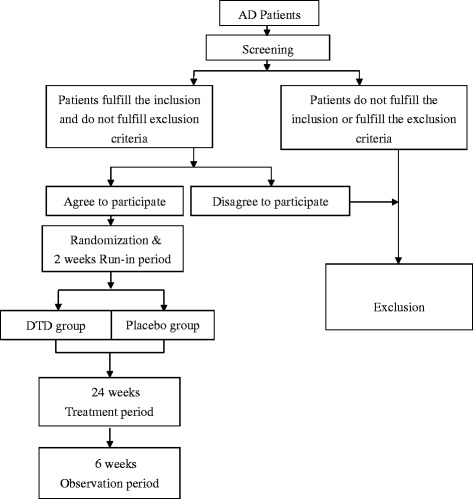
Schedule of enrollment.

Figure 1 shows the schedule of enrollment. We plan to recruit at least 40 patients, which will allow 20 for the DTD group and 20 for the placebo group in this study. All patients diagnosed with AD will be referred to a Chinese medicine doctor who is the Principle Investigator of the study (M. Li), or to a Research Assistant (K.K. Chua), for further assessment and recruitment. The aim, procedures, nature of study and possible side effects of DTD will be explained by the PI or RA; then, each subject will be asked to sign a written consent [see Additional file 4] to take part in the study. Patients will be informed that they are free to withdraw at any time during the study.

All patients will undergo a 2-week run-in period, in which they have to keep their Western medicine and supplement dosages fixed. Their blood will be tested to check liver function (alanine transaminase (ALT)/serum glutamic pyruvic transaminase (SGPT), aspartate transaminase (AST)/ serum glutamic oxaloacetic transaminase (SGOT), alkaline phosphatase, gamma glutamyltransferase, total bilirubin, total protein, albumin) and renal function (urea, creatinine, sodium, potassium, chloride, bicarbonate) in a lab test center designated for the project.

### Randomization and masking

The randomization sequence will be generated by SPSS 19.0 package (SPSS, Chicago, IL). The sequence will be password-protected and kept in a computer by L.L. Chen. Group allocation will be simple randomization in a ratio of 1:1 to either active treatment group or placebo group. The number sequences will be kept in sealed opaque envelopes and distributed to assessors. Patients, investigators and all sponsoring parties will be masked to treatment allocation until the end of the study. When there is a serious adverse event, the event will be discussed between the principal investigator Chinese medicine expert M. Li, and the co-investigator neurology specialist V. Mok to consider unblinding.

### Primary outcome and its assessment

A positive primary outcome of this study will be an increase of at least four points in the Alzheimer’s Disease Assessment Scale-cognitive subscale (ADAD-cog) [27] total score; this is considered to be a clinically meaningful increase [28]. Total scores of the ADAD-cog, Chinese version of Mini-Mental State Examination (C-MMSE) [29,30] and Chinese version of Disability assessment for dementia (C-DAD) score [31] will be used as secondary outcomes.

Outcome measurements will be carried out during the study visits at weeks 0, 12 (half of treatment), 24 (end of treatment) and 30 (end of observation period). Safety assessment, which includes reporting of adverse events (AEs), measurement of vital signs and physical examination, will be carried out throughout the study. In addition, laboratory safety screening of liver and renal function will be performed at week 24. Bilingual assessors, trained by the same neurology specialist V. Mok and clinical psychologist A. Wong, will be blind to the treatment allocation.

Clinical assessment will be rescheduled within 7 days after the scheduled clinical visit in case of any unexpected condition such as severe weather conditions. Phone call reminders will be given to the caregivers one day before the days of assessment.

A home diary will be given to each study participant’s caregiver to record treatment and changes in the participant’s medical condition. Formal instruction for the home diary will be given during the first visit. Revision and checking of the diary will be carried out with the caregiver during each formal visit by the assessors. Compliance in taking the treatments will be determined by the record of the diary and the number of the returned medicine/placebo packages.

### Data management and monitoring

All the data will be entered and stored in a password-protected computer. Hardcopy will be kept in a locker. No interim analyses will be carried out. To ensure high quality of the data, a double data entry method will be used. All documents and collected data will be kept for 7 years after the finish of study and then will be destroyed. The protocol and statistical results will be published in a scientific journal where the public will have access to all the results.

A data monitoring committee (DMC) will be formed and will include at least two faculty members from the participating universities, who are independent of the research team and who will not participate in any other aspect of the study. The data management process will be monitored by DMC regularly. All the data will be frozen and then locked to prevent further editing after the validation by the DMC. Only the DMC, the study research assistant and the principal investigator will have access to the final dataset.

### Statistical analysis

The proportion of patients with at least a four-point change in ADAS-cog and MMSE and the scores on C-DAD will be compared between the active treatment and placebo groups using the Chi-Squared test and independent sample t test, respectively. Missing data will be input using the last-observation-carried-forward (LOFC) approach. All patients randomized with at least one post-randomization measurement will be included in the primary analysis to follow the intention-to-treat principle. Analyses will be done with SPSS 19.0 package (SPSS, Chicago, IL).

To get a more comprehensive picture of the efficacy of DTD, a post-hoc analysis of the differences in the change of ADAS-cog and C-DAD sub-scores of the active treatment group will be compared between the active and placebo groups. To reduce the number of statistical comparisons, analyses will be performed with a hierarchical approach. To begin, the scores of ADAS-cog at week 24 (end of treatment) for the active treatment and placebo groups will be compared. If the difference is deemed statistically significant at a 2-sided *α*-level of 0.05, the scores of ADAS-cog at week 12 (half of treatment) and week 30 (end of observation period, that is, without treatment) will be compared between groups. C-DAD will be analyzed in the same manner as ADAS-cog.

### Compliance strategy

This will be a 30-week clinical trial, in which subjects will need to take study medication for 24 weeks with 8 regular visits (week 0, 4, 8, 12, 16, 20, 24 and 30). In order to maximize subjects’ compliance, first, we will have a thorough consent process for all participants; we will explain in detail the study schedule, potential side effects of treatment, and the responsibilities of the subjects. Second, we will screen potential subjects carefully during a 2-week run-in period in order to exclude ineligible patients or patients who are unlikely to comply with treatment regimen before randomization. Third, we will try to prevent dropouts by providing ongoing support to patients. A special e-mail account and a direct telephone line set up for this clinical trial will enable the study team to personally communicate with the patients. An information sheet will be given to each participant providing them and their caregivers with means of urgent contact. Extra visits and free medical care will be arranged for any participant who feels harmed by the trial protocol. Fourth, this is an add-on design [32], which means that the study treatment is added to their existing treatment. Certainly those participants receiving the placebo should not experience any difference in their condition, while those receiving the DTD should improve or at least remain the same.

### Early termination

The trial will be terminated for a specific participant in the event of any of the following: (1) participant develops severe adverse side effect(s); (2) participant shows hypersensitivity towards DTD; (3) participant develops some other life-threatening condition or disease; or (4) participant chooses to participate in another Chinese medicine research project. In addition, any participant may voluntarily withdraw.

The whole research plan will be terminated under the following circumstances: (1) presence of serious adverse effect(s) related to Chinese herbal medicine with supportive evidence or (2) completion of all follow-up assessments.

## Discussion

AD, as a progressive neurodegenerative disorder, has devastating impacts not only on patients but also on family members, friends, and caregivers. A cure would bring immeasurable relief and hope to countless thousands. Unfortunately, western pharmacological research has been unsuccessful in finding effective treatment without serious side effects [8]. In this case, Chinese medicine may have something to offer. Chinese medicine has been used to treat neurological diseases for thousands years with efficacy, safety, and relatively few side effects - albeit without rigorous and objective testing [33].

This randomized trial will be the first rigorous testing of DTD for the treatment of AD patients. Success in this clinical trial will provide evidence acceptable to the scientific community of the potential of DTD to become an effective medicine for AD. The success of this study would represent justification and impetus for a large-scale clinical trial to further consolidate the evidence for the use of DTD in the treatment of AD.

It is noted that the sample size calculation does not apply to this study. Normally, 10 to 20 patients in each group would be sufficient to implement the method of a pilot study [34]. Since there is no previous data indicating the sample size needed to unequivocally determine the effect of DTD by the ADAS-cog, the evidence will still be weak even if we have a positive result. Nevertheless, the one aspect of this study that could undermine its value is sample size. There is no information on what sample size is needed to give statistically significant results. On the other hand, this study could find out what specific symptoms, like mood or cognitive, that DTD may improve. This information should be an important evidence for a much precise clinical trial with larger sample size in the future.

In conclusion, the results of this study are expected to provide evidence for the efficacy and safety of the Chinese herbal formula, DTD, for treatment of AD patients who also meet the diagnostic criteria of “phlegm obstructing the orifices” syndrome according to TCM diagnostics. It may also provide a new direction for the clinical research on AD and TCM.

## Trial status

The trial has been approved and registered. At the time of manuscript submission, the study has been actively enrolling subjects, and so far has a total of 36 subjects. The study is still ongoing. Herbal and placement packets have been produced.

## Additional files

Additional file 1:
**SPIRIT-Checklist 2013.**
Additional file 2:
**Diagnostic criteria for “phlegm turbidity obstructing the orifices”(PTOO).**
Additional file 3:
**Test report of DTD quality control.**
Additional file 4:
**Informed consent statement of the Trial.**

## Abbreviations

Ach: acetylcholine
AchE: acetylcholine esterase
AD: Alzheimer’s disease
ADAD-cog: Alzheimer’s Disease Assessment Scale-cognitive subscale
AEs: adverse events
ALT: alanine transaminase
AST: aspartate transaminase
C-DAD: Chinese version of Disability Assessment for Dementia
CDR: Clinical Dementia Rating Scale
ChAT: acetylcholine transferase
ChEIs: cholinesterase inhibitors
C-MMSE: Chinese version of Mini-Mental State Examination
CONSORT: Consolidated Standards of Reporting Trials
DMC: Data monitoring committee
DTD: Di-tan decoction
LOFC: last-observation-carried-forward
NINCDS/ADARA: National Institute of Neurological and Communicative Disorders and Stroke and Alzheimer’s Disease and Related Disorders Association
PTOO: phlegm turbidity obstructing the orifices
SGOT: serum glutamic oxaloacetic transaminase
SGPT: serum glutamic pyruvic transaminase
SPIRIT: Standard Protocol Items: Recommendations for Interventional Trials
TCM: Traditional Chinese Medicine
