# Supplementary material for: The efficacy and safety of the Chinese herbal medicine Di-Tan decoction for treating Alzheimer’s disease: protocol for a randomized controlled trial
Source: Trials. 2015 Apr 30;16:199. doi: 10.1186/s13063-015-0716-z (PMC4426181; doi:10.1186/s13063-015-0716-z)
Supplement: Additional file 1: — SPIRIT-Checklist 2013. [file 13063_2015_716_MOESM1_ESM.docx]

**Additional file 1. Diagnostic criteria for “phlegm turbidity obstructing the orifices”(PTOO)**

**Diagnostic criteria for phlegm turbidity obstructing the orifices (PTOO):**

(1) Primary symptoms: dementia (MMSE<24); heavy-headedness; torpid intake; dribbling phlegm

(2) Secondary symptoms: mental sluggishness; overweight and obesity (BMI>25); heavy limbs; oppression in the abdominal; nausea and vomiting; lethargy; manic; thick tongue; slippery pulse.

For the diagnosis of PTOO, the AD patients must have at least 2 primary symptoms, plus 2 or more secondary symptoms.

Reference:

Xiao-Yu Zheng. *The Guidance for Clinical Research of New Chinese Herbal Medicine*. Beijing, Chinese Medicine Scientific Publisher, 2002. P95.
