# Supplementary material for: The efficacy and safety of the Chinese herbal medicine Di-Tan decoction for treating Alzheimer’s disease: protocol for a randomized controlled trial
Source: Trials. 2015 Apr 30;16:199. doi: 10.1186/s13063-015-0716-z (PMC4426181; doi:10.1186/s13063-015-0716-z)
Supplement: Additional file 2: — Diagnostic criteria for “phlegm turbidity obstructing the orifices”(PTOO). [file 13063_2015_716_MOESM2_ESM.pdf]

檢驗報告 Test Report

編號:No （W）20120593　　日期:Date 2012-8-28　　頁 Page 1/4

培力（南寧）藥業有限公司檢測中心 Purapharm(Nanning)Pharmaceuticals Co.,Ltd. Testing Laboratory
中國廣西南寧市高新技術開發區科園大道 46 號　No.46, Ke Yuan Road,Nanning New & High-tech Industrial Development Zone, Guangxi,China.

對“滌痰湯配方顆粒”之樣品之分析報告
Report on the submitted sample identified by the client as 滌痰湯配方顆粒

| | |
|---|---|
| 樣品名稱:Product Description | 滌痰湯配方顆粒 |
| 樣品編號: Product code | 3137 |
| 樣品規格:Product Specification | 13.5g/包 |
| 批號:Batch No. | A120704 |
| 流水號: Production running number | A120704 |
| 有效期至: Expiry Date | 2015 年 06 月 13 日 |
| 本批數量: Quantity produced | 1194 袋 |
| 樣品收到時狀態:Sample Receiving Condition | 室溫下存放於密封光身複合膜原來包裝中 |
| 製造商:Manufacturer | 生產部 |
| 委託檢驗單位: Inspected Entity | 生產部 |
| 來源地:Region of Origin | 生產部 |
| 目的地:Region of Destination | 培力（南寧）藥業有限公司檢測中心 |
| 檢驗日期:Testing Period | 2012 年 8 月 20 日至 2012 年 8 月 28 日 |

測試項目、分析方法及分析結果 Test Requested, Test Method and Test Results
請參考續頁。Please refer to the following page(s)

********

Signed for and on behalf of Purapharm(Nanning)Pharmaceuticals Co.,Ltd. Testing Laboratory
培力（南寧）藥業有限公司檢測中心代表簽名

096AA7694

檢驗報告 Test report

編號:No（W）20120593　　日期:Date2012-8-28　　頁 Page: 2/4

測試項目及分析方法 Test Requested and Test Method

| 測試項目 Test Items | 參考方法 Reference Method |
|---|---|
| 1. 性狀:Appearance | 中國藥典, 2010 年版, 第一部, 附錄 I C<br>The Pharmacopoeia of the People's Republic of China 2010, Vol.1, Appendix I C |
| 2. 水分含量: Determination of water | 中國藥典, 2010 年版, 第一部, 附錄IX H<br>The Pharmacopoeia of the People's Republic of China 2010, Vol.1, Appendix IX H |
| 3. 粒度 Size of granule | 中國藥典, 2010 年版, 第一部, 附錄XI B<br>The Pharmacopoeia of the People's Republic of China 2010, Vol.1, Appendix XI B |
| 4. 溶化性 Determination of dispersibility | 中國藥典, 2010 年版, 第一部, 附錄 I C<br>The Pharmacopoeia of the People's Republic of China 2010, Vol.1, Appendix I C |
| 5. 装量差异 Variation in content | 中國藥典, 2010 年版, 第一部<br>The Pharmacopoeia of the People's Republic of China 2010, Vol.1 |
| 6. 鑒別 Identification | 中國藥典, 2010 年版, 第一部, 附錄VI B<br>The Pharmacopoeia of the People's Republic of China 2010, Vol.1, Appendix VI B |
| 7.含量測定 Assay | 中國藥典, 2010 年版, 第一部, 附錄VI D<br>The Pharmacopoeia of the People's Republic of China 2010, Vol.1, Appendix VI D |
| 8. 微生物限度 Microbial Limit Test | 中國藥典, 2010 年版, 第一部, 附錄 XIII C<br>The Pharmacopoeia of the People's Republic of China 2010, Vol.1, Appendix XIII C |
| - 細菌數 Microbial count | |
| - 霉菌及酵母菌 Mould and Yeast Count | |
| - 大腸埃希菌 Escherichia coli | |
| 9. 重金屬 Heavy Metal Limit Test | 中國藥典 2010 年版一部附錄IX B；電感耦合等離子體質譜法；<br>The Pharmacopoeia of the People's Republic of China 2010, Vol.1, Appendix IX B；<br>ICP-MS |
| 10. 農藥殘留 Pesticides | 香港中醫藥管理委員會《中成藥註冊申請手冊》<br>Chinese Medicine Council of Hong Kong《Application Form: Registration of proprietary Chinese medicines》 |

********

096AA7695

檢驗報告 Test Report

編號:No（W）20120593 日期:Date2012-8-28 

分析結果 Test Results

| 測試項目 Test Items | 品質標準 Quality Specification | 滌痰湯配方顆粒 |
|---|---|---|
| 1. 性狀: Appearance | 顆粒劑應乾燥、顆粒均勻、色澤一致，無吸潮、軟化、結塊、潮解等現象。Granules should be dry, uniform in size and consistent in colour. There should be no moisture absorption, softening, clumping, deliquescence, etc. | 符合規定 Comply |
| 2. 水分含量 Determination of water | ≤6.0% (W/W) | 2.5% 符合規定 Comply |
| 3. 粒度 Size of granule | 不能通過一號篩和能通過五號篩的顆粒和粉末總和不得超過 15% Sum of weight of granules that cannot pass through sieve no.1 and weight of powder that can pass through sieve no.5≤15% | 4% 符合規定 Comply |
| 4. 溶化性 Determination of dispersibility | 允許有輕微渾濁，並均不得有焦屑等異物 Light turbidity is allowed and no foreign matters | 符合規定 Comply |
| 5. 裝量差异 Variation in content | 應符合規定 Shall meet the requirement | 符合規定 Comply |
| 6. 鑒別 Identification | 應符合規定 Shall meet the requirement | 符合規定 Comply |
| 7.含量測定 Assay | 以橙皮苷计，不得少於 1.87mg/g Hesperidin no less than1.87 mg/g | 3.14mg/g 符合規定 Comply |
| 8. 微生物限度# Microbial Limit Test | | |
| -細菌數 Microbial count | ≤500 cfu/g | 40 cfu/g 符合規定 Comply |
| -霉菌及酵母菌 -Mould and Yeast Count | ≤100 cfu/g | <10 cfu/g 符合規定 Comply |
| - 大腸埃希菌 Escherichia coli | 不得檢出/克 Absent /g | 未檢出/克 Absent /g 符合規定 Comply |
| 9.重金屬 Heavy Metal Limit Test | 銅(Cu)不得過 150mg/kg | 1.449mg/kg 符合規定 Comply |
| | 砷(As)不得過 41.67mg/kg 或 1500μg/日 | 0.276mg/kg(相當于 9.94μg/日) 符合規定 Comply |
| | 鎘(Cd)不得過 97.22mg/kg 或 3500μg/劑 | 0.016mg/kg(相當于 0.58μg/劑) 符合規定 Comply |
| | 鉛(Pb)不得過 4.97mg/kg 或 179μg/日 | 0.189mg/kg(相當于 6.80μg/日) 符合規定 Comply |
| | 汞(Hg)不得過 1.00mg/kg 或 36μg/日 | 0.001mg/kg(相當于 0.04μg/日) 符合規定 Comply |
| 10. 農藥殘留 Pesticides | 艾氏劑及狄氏劑 Aldrin & Dieldrin 兩者之和≤0.05 mg/kg | 未檢出 符合規定 Comply |
| | 氯丹 Chlordane Cis, trans- 異構體與 oxychlordane 之和≤0.05 mg/kg | 未檢出 符合規定 Comply |
| | 滴滴涕 DDT P, p'-DDT, o,p'-DDT, p,p'-DDE 與 p,p'-TDE 之和≤1.0 mg/kg | 未檢出 符合規定 Comply |
| | 異狄氏劑 Endrin Endrin≤0.05 mg/kg | 未檢出 符合規定 Comply |
| | 七氯 Heptachlor Heptachlor 與 heptachlor epoxide 之和≤0.05 mg/kg | 未檢出 符合規定 Comply |
| | 六氯苯 Hexachlorobenzene Hexachlorobenzene≤0.1 mg/kg | 未檢出 符合規定 Comply |
| | 六六六 Hexachlorocyclohexane α-BHC，β-BHC，δ-BHC 異構體之和≤0.3 mg/kg | $1.72\times10^{-3}$mg/kg 符合規定 Comply |
| | 林丹 Lindane Lindane≤0.6 mg/kg | 未檢出 符合規定 Comply |
| | 五氯硝基苯 Quintozene Quintozene, pentachloroaniline 與 methyl pentachlorophenyl sulphide 之和≤1.0 mg/kg | 未檢出 符合規定 Comply |

*********

096AA7696

PuraPharm 培力

TESTING
CNAS L4059



註：Remarks

1、 報告無“檢驗/檢測報告專用章”和缺騎縫章無效。

Test report without the stamp of “For Test/Test Report Only” is not authorized.

2、 複製的報告未重新加蓋“檢驗/檢測報告專用章”及騎縫章無效。

Certified copy of test report without the stamp of “For Test/Test Report Only” is not authorized.

3、 報告無編制、審核及本中心代表簽字無效。

Test report must be authorized by the signature of the bodies of preparation, verification and representative of the testing centre.

4、 報告塗改、缺頁無效。

Test report with modification or lacking of page(s) is not authorized.

5、 對報告若有異議，請於收到報告之日起十五日內向檢驗單位提出書面申訴，否則按認可檢驗報告處理。本中心異議受理電話 0771-3218026。

The client is aggrieved by the test result. A request for review shall state in writing the reasons relied upon and shall be made to the testing centre within 15 days after receipt of the authorized test report. No requirement will be accepted after the definite time. Inquiry hot line is 0771-3218026.

6、 送樣委託檢驗，樣品名稱為委託單位自報名稱，報告僅對來樣負責。部份複製檢驗/檢測報告無效。

Name of tested product is provided by the client. The report will refer only to the sample tested. Copy of partial of the test report is not authorized.

# - 其他微生物限度要求: Other requirements for Microbial Limit Test

1.霉菌及酵母菌: 固體製劑及不含糖、王漿、蜂蜜的液體製劑或半固體製劑檢查霉菌；含糖、王漿、蜂蜜的液體製劑或半固體製劑檢查霉菌和酵母菌。

A)Mould and Yeast Count: For dose in solid form and liquid form not containing sugar, royal jelly, honey or semi-solid form, mould count should be tested. For dose in liquid form containing sugar, royal jelly, honey or semi-solid form, both mould count and yeast count should be tested.

2.含有動物類原藥材的口服製劑，不得檢出沙門氏菌；含動物角、王漿、蜂蜜、阿膠的口服製劑，則毋須進行有關檢定。

For oral preparations containing animal-origins raw ingredients, Salmonella should not be detected. For oral preparations containing animal horn, royal jelly, bee honey and Colla Corii Asini, test for Salmonella may be exempted.

*** End of Report 報告完 ***

096AA7697

PuraPharm (Nanning) Pharmaceuticals Co., Ltd. Testing Laboratory 培力（南寧）藥業有限公司檢測中心
No. 46 Keyuan Road, Nanning New and High-tech Industrial Development Zone, Nanning, Guangxi, China 中國廣西南寧市高新技術開發區科園大道 46 號
Tel 電話：(86) 771-321-8026 • Fax 傳真：(86) 771-321-6602 • Website 網址：www.purapharm.com
