# Supplementary material for: The efficacy and safety of the Chinese herbal medicine Di-Tan decoction for treating Alzheimer’s disease: protocol for a randomized controlled trial
Source: Trials. 2015 Apr 30;16:199. doi: 10.1186/s13063-015-0716-z (PMC4426181; doi:10.1186/s13063-015-0716-z)
Supplement: Additional file 4: — Informed consent statement of the Trial. [file 13063_2015_716_MOESM4_ESM.doc]

**Additional file 4. Informed consent statement**

**HONG KONG BAPTIST UNIVERSITY**

**INFORMED CONSENT STATEMENT**

**A Randomized, Double-blind, Placebo-controlled Clinical Trial of**

**Chinese Medicine** **(Di-Tan Decoction) for the Treatment of Alzheimer’s disease**

You are invited to participate in a research study. The purpose of this study is to evaluate the efficacy and safety of Chinese herbal medicine (Di Tan Decoction) in Alzheimer’s disease, and its impact on the quality of life. Before you decide to take part in this study, please make sure you have fully understood the purpose as well as important related things about the research. Please think it deeply before your decision.

**INFORMATION**

Alzheimer’s disease (AD) is one of the popular brain degenerative diseases in human. Traditional Chinese medicine (TCM) has been used to treat neurological diseases for thousands of years with efficacy and resonable safety. According to our recent literature review, there are several animal studies suggested that Di-Tan Decoction (DTD) is effective in improving the cognitive function of AD rats. It is also listed in the unified Chinese Medicine textbook for treating dementia in 2007. However, it still requires rigorous scientific evidence from clinical trials that show direct correlation. Hence, the aim of this research is to observe and verity the efficacy of DTD in treating AD by a rigid randomized, double-blind, placebo-controlled clinical trial.

Based on the inclusion and exclusion criteria, participants diagnosed with “phlegm turbidity obstructing the orifices” under TCM rationale are recruited for a 30-week clinical trial. This involves 2-week run-in period, 24-week treatment period and 6-week observation period. Before and after the medication, participants may have liver and kidney function tests to ensure normal function as well as the safety of the research. Additional blood test may be provided if it is needed during the treatment. After the run-in period, suitable participants will be randomized to receive either DTD or placebo granule with concurrent western medications. Patients may return to the Chinese Medicine outpatient service clinic of Hong Kong Baptist University regularly with total 8 visits at 0-week, 4-week, 8-week, 12-week, 16-week, 20-week, 24-week and 30-week. Each may last for about 0.5 to 1.5 hours. Besides, a self-complete daily will be distributed to the participants to monitor their health. This is a doubt-blinded, randomized, placebo-controlled clinical trial. Neither the registered Chinese Medicine practitioner nor the participants knew the allocation during the whole research period. The placebo, with no active ingredients would be used for clinical control.

**RISK**

There is currently no known side-effect of DTD granule. However, this cannot be guaranteed. A small number of people may experience adverse events such as skin allergic reaction, diarrhea, vomiting and the like. In case it happens, the clinical trial will be stop immediately and further medical treatment or referral will be given if necessary.

**EMERGENCY MEDICAL TREATMENT**

In the unlikely event of physical injury resulting from your participation in this research, emergency medical treatment will be provided at no cost to you. Be certain that you immediately notify the investigators if you are injured. If you request additional medical treatment, you will be responsible for the cost. No other compensation will be provided for injuries.

**BENEFITS**

All registration fee and treatment fee will be exempted for patients attending this trial. HKD1,000 traveling subsidy will be given after completed the whole trial. Though taking part in this research may or may not be beneficial the individual, but it will be helpful to find a treatment for Alzheimer’s disease. However, your participation in TCM research would definitely makes contribution on the further researches about AD and Traditional Chinese Medicine in the future.

**CONFIDENTIALITY**

Every participant will be randomly assigned a code which can be traced back to the group allocation. The code list will store in both hard and soft copies. The hard copy will be stored in a tray with key lock while the soft copy will be saved with password. People other than the research team are not allowed to assess these data. Questionnaires, patient diaries and related document will be kept entirely confidential and only the research team member has rights to read them. The reports will be written mainly in aggregate term, but individual responses to drug may also be described and no personal data will be disclosed in any report. All documents and collected data will be kept for 7 years and then will be destroyed. All the information provided by the participant will be kept confidential by the research team.

**COMPENSATION**

If you get any other disease during the research period, no compensation will be provided for it. If you have direct injury due to the research, you can apply for compensation with enough and strong evidence. On the other hand, if you feel you have not been treated according to the descriptions in this form or your rights as a participant in research have been violated during the course of this project, you may contact the Committee on the Use of Human and Animal Subjects in Teaching and Research of the University at hasc@hkbu.edu.hk.

**CONTACT**

If you have questions about the study or the procedures, you may contact the registered Chinese Medicine practitioner, Mr. Chua Ka Kit.

Address: Teaching Division, School of Chinese Medicine, Hong Kong Baptist University, Kowloon Tong, Hong Kong.

Telephone: 6700-8538

Email: bucmad@hkbu.edu.hk

**PARTICIPATION**

Your participation in this study is completely voluntary; you may decline to participate without penalty. If you decide to participate, you may withdraw from the study at any time without penalty and without loss of benefits to which you are otherwise entitled. If you withdraw from the study before data collection is completed, your data will be destroyed.

**CONSENT**

I have read and understand the above information and given the chance if I question. I agree to participate in this study and would be given a copy of this form.

＿＿＿＿＿＿＿＿＿＿＿＿＿　　＿＿＿＿＿＿＿＿＿＿＿＿＿　　＿＿＿＿＿＿＿＿＿＿＿

Name of participant/proxy　　 Sign of participant/proxy　　　　 Date

＿＿＿＿＿＿＿＿＿＿＿＿＿　　＿＿＿＿＿＿＿＿＿＿＿＿＿　　＿＿＿＿＿＿＿＿＿＿＿

Name of witness　　　　　 Sign of witness　　　　　　 Date

＿＿＿＿＿＿＿＿＿＿＿＿＿　　＿＿＿＿＿＿＿＿＿＿＿＿＿　　＿＿＿＿＿＿＿＿＿＿＿

Name of investigator　　　　 Sign of investigator　　 　　　 Date

－End of Informed Consent Statement－
